# Psychometric Validation of the Arabic FRAIL Scale for Frailty Assessment Among Older Adults with Colorectal Cancer

**DOI:** 10.3390/healthcare13233117

**Published:** 2025-12-01

**Authors:** Mohammed T. A. Omar, Bader Nasser M. Alamri, Ahmed Mohammed Mesfer, Majed Hassan Al-Malki, Ahmed Allehebi, Zizi M. Ibrahim, Rehab F. M. Gwada

**Affiliations:** 1 Rehabilitation Health Sciences Department, King Saud University, P.O. Box 10219, Riyadh 11344, Saudi Arabia; rgwada@ksu.edu.sa; 2 Faculty of Physical Therapy, Cairo University, Giza P.O. Box 12612, Egypt; 3 Physiotherapy Department, Eradah Mental Health Complex, Al-Montazah, Dammam 34242, Saudi Arabia; shoot.ops1@gmail.com; 4 Medical Rehabilitation and Long-Term Care, Al-Baha Health Cluster, Riyadh 12811, Saudi Arabia; aalzahrani284@moh.gov.sa; 5 Medical Rehabilitation Department, Prince Mishari Bin Saud Hospital, Olaya 11564, Saudi Arabia; 6 Medical Oncology Department, King Faisal Specialist Hospital and Research Center, Jeddah 23433, Saudi Arabia; 7 Department of Rehabilitation Sciences, College of Health and Rehabilitation Sciences, Princess Nourah bint Abdulrahman University, P.O. Box 84428, Riyadh 1167, Saudi Arabia; zmibrahim@pnu.edu.sa; 8 Physical Therapy Department, National Heart Institute, Giza 12613, Egypt

**Keywords:** colorectal cancer, FRAIL scale, validity, frailty

## Abstract

**Highlights:**

**What are the main findings?**

- The FRAIL-AR scale demonstrated good internal consistency and good test–retest reliability.
- FRAIL-AR scores in elderly Colorectal Cancer patients significantly correlated with function, as well as their quality of life.

**What is the implication of the main findings?**

- The FRAIL-AR is a reliable and culturally appropriate tool enabling geriatricians and oncologists to perform rapid frailty screening in Arabic-speaking older adults with Colorectal Cancer.
- Implementation of the validated FRAIL-AR scale can support personalized treatment planning and the development of targeted interventions to ultimately improve outcomes for CRC patients.

**Abstract:**

Background/Objective: Culturally adapted frailty screening tools are essential for improving health outcomes, facilitating clinical decision-making, promoting effective care planning, and ensuring accurate frailty assessment across diverse cultural contexts; their use among clinicians and academics is therefore supported. The purpose of this study was to assess internal consistency, test–retest reliability, and validity of the Arabic FRAIL scale (FRAIL-AR scale) for Arabic-speaking populations with colorectal cancer (CRC). Methods: This cross-sectional study included 137 participants diagnosed with CRC who completed the FRAIL-AR scale, the EORTC QLQ-C30 physical function subscale, and functional performance-based Timed Up and Go (TUG) and Five Times Sit-to-Stand (5xSTS) tasks. Internal consistency was assessed using Kuder–Richardson formula 20 (KR-20), and test–retest reliability was determined using the two-way random intraclass correlation coefficient ICC _(2.1)_. Convergent validity was evaluated by assessing the correlation between the FRAIL-AR scale against the EORTC QLQ-C30 physical function scale, TUG, and 5xSTS. Results: The FRAIL-AR scale exhibited good internal consistency (KR-20 = 0.80) and test–retest reliability (ICC _(2.1)_ = 0.89, 95% CI 0.77–0.94). Correlation analysis showed a weak negative correlation between the overall FRAIL-AR scale scores and EORTC QLQ-C30 physical function scale scores (*r* = −0.38, *p* < 0.05), while it exhibited a moderate positive correlation with TUG (*r* = 0.75, *p* < 0.01) and 5xSTS (*r* = 0.63, *p* < 0.01) scores. FRAIL-AR scores showed significant known-groups validity with higher frailty scores in older-age individuals (*p* < 0.01), females (*p* < 0.05), and those with comorbid conditions (≥5) (*p* < 0.05). Conclusion: The FRAIL-AR scale’s validity and reliability make it an appropriate tool for geriatricians, oncologists, and healthcare providers to evaluate and monitor frailty among Arabic-speaking colorectal cancer patients.

## 1. Introduction

### Background

Colorectal cancer (CRC) is a significant health issue and the third most common cancer worldwide [1]. Its incidence increases with age, and 90% of CRC patients are at least 50 years old [2]. Managing CRC often requires intensive treatment such as surgery and chemotherapy, which can diminish physiological reserves and increase the risk of frailty, with a reported incidence rate of up to 60.5% [3]. Frailty is a critical factor in CRC management, correlating with an increased risk of mortality, fall-related injuries, immobility, and functional decline, which often leads to long hospital stays and delayed bowel function recovery [3,4]. It also affects chemotherapy tolerance, increasing the risk of toxic effects and recurrent admissions and significantly affecting quality of life [5,6]. Therefore, early frailty screening is essential to understand the health status of vulnerable cancer patients, guiding cancer treatment decisions and supportive care to improve health-related outcomes [7].

Several frailty assessment instruments are available with different operational definitions: physical, biological, psychological, and social functioning [8,9]. However, a consensus on the best screening tool for frailty has not been reached, and there is no gold-standard instrument [7,8,9,10]. In 2007, an international panel developed the FRAIL scale as a self-report tool derived from the “phenotypic model of frailty.” It measures frailty across five domains: fatigue, resistance, ambulation, illnesses/comorbidities, and weight loss [11,12]. Because of its simplicity, rapidity, low cost, and capacity for self-reporting, the FRAIL scale has been used in a variety of clinical settings and research studies. It also has cultural adaptability among different populations, regions, and concomitant disorders [13,14,15,16,17,18,19,20,21,22] and has shown utility in assessing vulnerability and predicting adverse outcomes across multiple oncological contexts, including hematologic malignancies and various solid tumors; however, its psychometric properties need dedicated verification in the CRC population [13,15]. CRC is strongly associated with factors that directly influence the core components of the FRAIL score. CRC is often characterized by chronic inflammation, malabsorption, and tumor-induced metabolic changes, which frequently lead to significant unintentional weight loss and profound fatigue [23,24]. Furthermore, disease progression and treatment often result in muscle mass deterioration (sarcopenia) and an associated decline in physical performance, making activities like walking difficult [24,25]. Validation of the FRAIL scale in these patients would permit a rapid and accurate assessment of frailty, guiding timely clinical decisions, personalization of oncologic treatment, and strategic rehabilitation planning to promote better health outcomes and quality of life [26,27].

The elderly population in Saudi Arabia is predicted to rise to 22.9% by 2050. This demographic shift is a major healthcare issue due to the higher burden of chronic diseases, including cancer and frailty [28]. The prevalence of frailty varies significantly among older people in Saudi Arabia, ranging from 21% to 40% and over 50% for community-dwelling elderly and hospitalized old adults, respectively [20,21,22,23,24,25,26,27,28,29,30,31,32]. This prevalence is expected to increase due to the substantial incidence of CRC (14.6%) [33].

Culturally adapted frailty screening tools are essential for improving health outcomes, facilitating clinical decision-making, promoting effective care planning, and ensuring accurate frailty assessment across diverse cultural contexts, thus supporting their use among clinicians and academics [34,35]. Several frailty tools such as the FRAIL scale, the Tilburg Frailty Indicator, and the Groningen Frailty Indicator have been adapted and validated for the Arabic-speaking population. However, their validity for older adults with CRC is not yet supported. Therefore, the purpose of this study was to assess the internal consistency, test–retest reliability, and validity of the Arabic FRAIL scale in older participants with colorectal cancer.

## 2. Materials and Methods

### 2.1. Setting and Participants

In this cross-sectional observational study, participants were recruited from the medical oncology outpatient clinic at King Faisal Specialist Hospital Research Center in Jeddah (KFSHRC-J), Saudi Arabia. This study represents a sub-analysis using baseline measurements of association between physical function and HRQOL in older adult CRC patients with or without frailty. It was approved by the Institutional Review Board (IRB) of JFKFSH&RC-J on 25 October 2021, and assigned the reference number IRB 2021-81. Ethics approval was valid for one year, fully covering the participant recruitment period from October 2021 to March 2022. All participants provided written informed consent prior to enrollment.

Participants were eligible if they were 60 years or older, diagnosed with CRC, had survived at least six months after treatment (e.g., surgery, chemotherapy, or radiotherapy), and were able to read and understand Arabic. Patients with multiple recurrent tumors, in palliative care, with hearing or vision impairment, or with other severe life-threatening conditions were not enrolled.

### 2.2. Sample Size

The sample size was calculated with G*Power software 3.1.9.2 [36]. To establish validity, we used a correlation coefficient of 0.3 between the FRAIL scale and the EORTC QLQ-C30 physical function subscale. Using an alpha level of 0.05 and a power of 0.80, a minimum of 84 participants were required. The target sample size was increased to 100 participants in line with the recommendation of the COSMIN guidelines for validation studies [37]. Considering an attrition rate of about 20%, the sample size was 120.

### 2.3. Outcome Measures and Procedure

Oncologists referred participants with CRC to physical therapists for eligibility assessment and to obtain informed consent. Demographic data such as age, gender, marital status, education level, employment, and residence were recorded via face-to-face interviews. Medical history including tumor stage, treatment history, and health-related comorbidity was obtained from medical charts. Comorbidities were also estimated using the Charlson comorbidity index (CCI). The CCI includes 19 specific medical conditions and generates an index risk score from 0 to 33 [38]. Patients were grouped as mild (1–2), moderate (3–4), or severe (≥5) according to their CCI scores [38]. At the initial visit, the physical therapist summarized the procedures and answered questions from the participant, after which tests were performed in a consistent order to minimize potential fatigue effects. The tests were performed in the standardized order: (1) FRAIL scale; (2) patients’ health-related quality of life measured using the European Organisation for Research and Treatment of Cancer Quality of Life Questionnaire-Core 30 (version 3.0) (EORTC QLQ-C30); (3) Timed Up and Go (TUG); and (4) Five-Times Sit-to-Stand (5xSTS) tests. Adequate rest of 2 min was provided between the two physical performance tests (TUG and 5xSTS). A convenience sample of 30 participants was assessed during a second visit within a 7-day period to estimate the test–retest reliability of the FRAIL scale. Trained research assistants verified the completeness of all responses before participants concluded the session to minimize the risk of missing item-level data.

#### 2.3.1. Five-Point Indicators Frailty Scale

The FRAIL scale is a self-reported scale with five domains that assess fatigue, resistance, ambulation, illnesses, and weight loss. Each of the five domains is scored from 0 to 1, with 1 indicating the presence of frailty. The total scores range from 0 to 5, with higher scores indicating greater frailty. Participants were grouped based on the FRAIL scale into frail (score ≥ 3 points), pre-frail (1–2 points), and non-frail subjects (0 points) [11,12].

#### 2.3.2. European Organisation for Research and Treatment of Cancer Quality of Life Questionnaire-Core 30 (Version 3.0)

The EORTC QLQ-C30 is a cancer-specific quality-of-life scale developed by the EORTC QOL research group that is widely used in clinical practice, as well as for quality-of-life trials among cancer patients [39]. It is a 30-item questionnaire with five functional scales—physical (5 items), role (2 items), cognitive (2 items), emotional (4 items), and social (2 items); three symptom scales (fatigue, pain, and nausea/vomiting); and six single-item symptom scales (dyspnea, sleep disturbance, appetite, diarrhea, constipation, and financial difficulties), together with a global health status/QOL item. All EORTC QLQ-C30 scales and single items were scored and linearly transformed to a 0–100 scale. Higher functional scale scores reflect a better level of functioning, while higher symptom scale scores are indicative of greater problems [39]. The Arabic version of the EORTC QLQ-C30 was found to have sufficient face and content validity, good predictive and concurrent validity, and internal consistency [40,41,42].

#### 2.3.3. Timed up and Go (TUG)

The Timed Up and Go (TUG) test measures physical performance, lower limb mobility, and strength in both clinical and research settings. Using a handheld stopwatch, TUG measures the time (in seconds) required for a participant to stand up from sitting in a chair with a seat height of 47 cm and an armrest, walk 3 m, turn around, walk back, and sit down again. The test was performed according to the standard procedure [43] with consistent verbal instructions for each participant to arise, walk 3 m as quickly as possible, and sit down again [43]. The test–retest reliability and validity of TUG have been documented in patients with cancer [44,45,46,47].

#### 2.3.4. Five Times Sit to Stand Test (5xSTS)

The 5xSTS test measures balance and functional strength [48]. Participants were asked to stand up from a chair and sit back down five times as fast and safely as possible. Participants’ arms were crossed over their chests with hands placed on shoulders. The duration (in seconds) was measured from the onset of the “start” command to when participants laid their backs against the backrest of the chair after completing 5 repetitions [49,50]. The 5xSTS test has demonstrated test–retest reliability and construct validity among older cancer survivors [51].

### 2.4. Statistical Analysis

Statistical Package for Social Sciences (SPSS) version 26 (IBM, SPSS Inc., Cary, NC, USA) was used to analyze the data. Normality tests indicated that the data were not normally distributed, and therefore, nonparametric tests were used. Means, frequencies, percentages, and standard deviations were calculated for all variables, including demographic and clinical characteristics and other outcome variables. *p* < 0.05 was considered statistically significant.

#### 2.4.1. Reliability Analysis

The internal consistency of the FRAIL-AR scale was evaluated using the Kuder-Richardson formula 20 (KR-20), with KR-20 ≥ 0.70 indicates acceptable internal consistency [52].

$$(\mathrm{KR}-20)=\left(\frac{K}{K-1}\right)\left(1-\frac{\sum pq}{\sigma^{2}}\right)$$

where *K* is the total number of questions, *p* is the proportion of yes answers, *q* is the proportion of no answers, ∑*pq* is the sum of *pq*, and *σ*^2^ is the total respondent’s variance.

Spearman correlation coefficients were calculated to estimate the association between individual item scores and overall scale score [53], categorizing the correlation as very weak (<0.20), weak (0.20–0.39), moderate (0.40–0.59), strong correlation (0.60–0.79), and very strong (>0.80) [53].

The test–retest reliability of the FRAIL-AR scale was established using intraclass correlation coefficients (ICC_2.1_) from a two-way random-effects model on absolute agreement for single measures, with 95% confidence intervals (95% CI). The following values are utilized to interpret the ICC results: values < 0.5 (poor), from 0.50 to 0.74 (moderate), 0.75 to 0.90 (good), and >0.90 (excellent) [54]. Absolute reliability was evaluated by calculating the standard error of measurement (SEM) and the smallest detectable change (SDC) [55].

#### 2.4.2. Floor and Ceiling Effects

Floor and ceiling effects were assessed by determining the proportion of patients with the lowest (FRAIL-AR score of 0) and highest (FRAIL-AR score of 5). Floor and ceiling effects below 15% are considered acceptable [56].

#### 2.4.3. Validity Analysis

For the convergent validity analysis, the Spearman correlation coefficient was used to evaluate hypothesized correlations of FRAIL-AR against the physical function subscale of EORTC QLQ-C30, the TUG test, and the 5xSTS. The correlation values are interpreted as following: very weak (<0.20), weak (0.20–0.39), moderate (0.40–0.59), strong (0.60–0.79), and very strong (>0.80) [53]. The correlation values are interpreted as follows: very weak (<0.20), weak (0.20–0.39), moderate (0.40–0.59), strong (0.60–0.79), and very strong (>0.80) [53]. We formulated and tested the hypotheses regarding the overall scores of the FRAIL -AR scale and its five domains; fatigue, resistance, ambulation, illness, and weight loss (Table 1).

Known-group validity was evaluated by analyzing the correlations of the FRAIL-AR scale with age, sex, and comorbidity. The following hypotheses were formulated and tested:

- FRAIL-AR scores will be significantly greater among CRC patients ≥ 75 years compared to those aged 65–74 years.
- Among older CRC patients, women will have higher FRAIL scores than men.
- Among older adult CRC patients, those with a severe CCI score (≥5) will demonstrate greater FRAIL-AR scores in comparison to those with a moderate CCI (3–4).

The effect size (Cohen’s D) was calculated as the difference in the mean/pooled standard deviation, with values of 0.2 generally considered “small,” 0.5 “medium,” and 0.8 “large.”

A total of 21 hypotheses (Table 1) were formulated to assess both construct and known-group validity. Confirmation of ≥75% of these hypotheses was considered as evidence supporting the sufficient validity of the FRAIL-AR scale [57].

Data completeness was checked following collection. All items across the FRAIL-AR scale and all other outcome measures had complete data; no missing values were observed (*n* = 137). Therefore, no imputation methods were required, and all analyses were performed on the full dataset.

## 3. Results

### 3.1. Participants Characteristics

**Figure 1:**
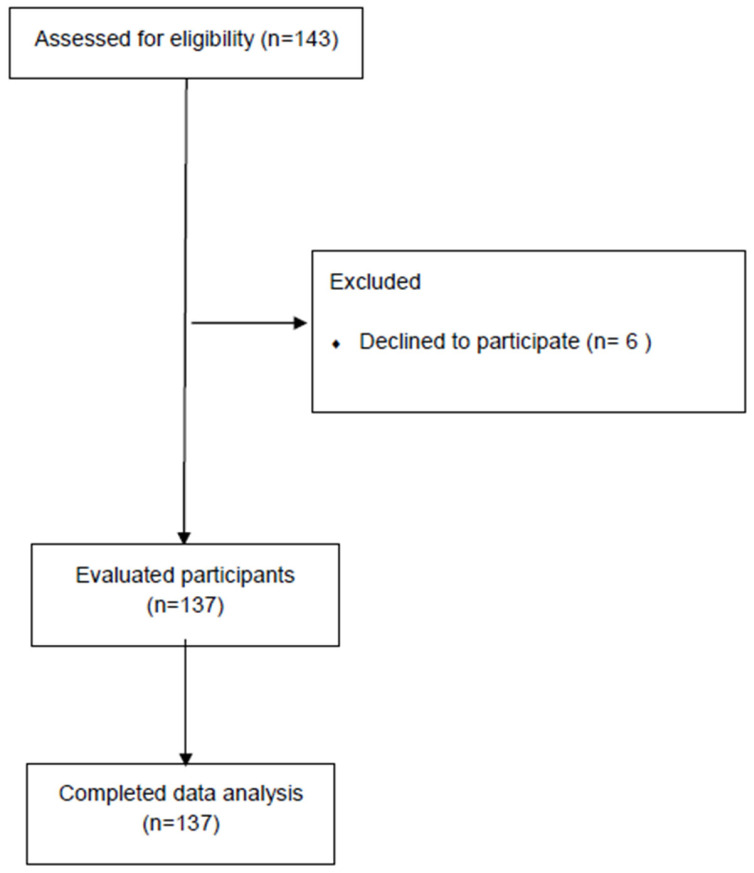
Flow chart for study.

Initially, 143 participants were recruited from the outpatient medical oncology center at KFSHRC in Jeddah. As shown in Figure 1, 6 patients were excluded as they refused to participate and 137 patients were included in the final analysis. Table 2 describes the demographic and clinical characteristics of all participants according to age group. The mean age of participants was 68.04 ± 6.99 years; most were aged 60–74 years (73.70%), male (62.80%), and married (75.20%). Approximately one-third (32.80%) had a university or higher degree, and over half (56.90%) were classified as overweight or obese. The majority were non-smokers (78.80%), and the average cancer duration was 28.03 ± 10.46 months. Colon cancer was predominant (65.70%), with the more than half of participants presenting tumor stages III–IV (54.74%). The most common treatment regimen combined surgery, chemotherapy, and radiotherapy (40.10%), and most participants demonstrated moderate (58.40%) or severe (41.60%) comorbidity risk.

### 3.2. Reliability

The total FRAIL-AR Scale score demonstrated good internal consistency with a KR-20 coefficient of 0.80 (95% CI: 0.73–0.85), exceeding the acceptable threshold of 0.70. Analysis of the item-to-total score correlation demonstrated strong correlations for the fatigue (*r* = 0.67, 95% CI; 0.49–0.71), resistance (*r* = 0.71, 95% CI; 0.58–0.76), ambulation (*r* = 0.71, 95% CI; 0.61–0.78,), and weight loss domains (*r* = 0.60, 95% CI; 0.50–0.79), while the illnesses domain showed a moderate correlation (*r* = 0.48, 95% CI; 0.45–0.71), (Table 3).

Test–retest administration occurred within a 7-day period, with a mean duration of 4.0 ± 2.5 days. The overall FRAIL-AR scale score demonstrated good test–retest reliability with ICC _(2.1)_ = 0.89 (95% CI: 0.77- 0.94). The illnesses domain showed excellent test–retest reliability, with ICC _(2.1)_ = 0.94 (95% CI: 0.87–0.97). Resistance (ICC _(2.1)_ = 0.89, 95% CI; 0.79–0.95), fatigue (ICC _(2.1)_ = 0.85, 95% CI; 0.71–0.93), and weight loss (ICC _(2.1)_ = 0.80, 95% CI; 0.62–0.90) presented good test–retest reliability, while ambulation showed moderate reliability with ICC _(2.1)_ = 0.71 (95% CI: 0.48–0.85) (Table 3). SEM for the overall FRAIL-AR scale score was 0.59, while SDC was 1.63.

### 3.3. Validity

Table 4 displays convergent validity of the FRAIL-AR Scale with EORTC QLQ-C30 physical function scale, TUG and 5xSTS scores. The overall FRAIL-AR scale score, along with specific five domains measuring fatigue, resistance, ambulation ability, and weight loss, showed a weak negative significant correlation (*r* = −0.22 to −0.38, *p* < 0.05) with EORTC QLQ-C30 physical function scores. While, the ‘illness’ domain demonstrated very week correlation (*r* = −0.15, *p* < 0.05). The overall FRAIL-AR score had a strong positive significant correlation with the TUG (*r* = 0.75, *p* < 0.01), while the five domains showed moderate correlations ranging from (*r* = 0.4 to 0.53, *p* < 0.01). The overall FRAIL-AR score had a strong positive significant correlation with the 5xSTS score (*r* = 0.63, *p* < 0.01), while the five domains showed moderate correlations ranging from (*r* = 0.4 to 0.46, *p* < 0.01). The FRAIL-AR scale had no ceiling issues given (14%) reached the maximum score while 40% participants reached the minimum score.

The known-group validity of the FRAIL-AR scale is shown in Table 5. Overall, the FRAIL-AR scale significantly differentiates between groups known to have different expected levels of frailty according to age, gender, and comorbidity severity. The comparison based on age group shows a statistically significant difference in the distribution of FRAIL-AR categories (*p* < 0.01). Pairwise comparison revealed that a significantly higher proportion of participants aged >75 years (58.34%) were classified as frail compared to the 65–74 age group (32.70%). Conversely, a higher proportion of the 65–74 age group were classified as robust (38.60% vs. 19.44%).

The comparison based on gender shows a statistically significant difference in the distribution of FRAIL-AR categories (*p* < 0.05). A significantly higher percentage of female participants (47.10%) were classified as frail compared to male participants (34.88%), and a higher percentage of males were classified as robust compared to females (36.05% vs. 23.50%). The comparison based on CCI scores shows a statistically significant difference in the distribution of FRAIL-AR categories (*p* < 0.05). A significantly greater percentage of individuals with severe CCI (>5) scores (54.40%) were classified as frail compared to those with moderate CCI scores (36.20%). A significant and substantial difference was found across the age, gender, and severe CCI (>5) groups, with respective effect sizes of 0.38 (95% CI; 0.1–0.67), 0.45 (95% CI; 0.15–0.84), and 0.49 (95% CI; 0.1–0.79) (*p* < 0.05). FRAIL-AR demonstrated strong convergent and known-group validity, as 20 (95%) of the pre-specified hypotheses were accepted (Table 1).

## 4. Discussion

To the best of our knowledge, this study is the first to validate the Arabic version of the FRAIL scale as a clinical frailty screening tool for elderly CRC patients. Our findings demonstrate strong internal consistency and test–retest reliability, while validating construct and known-group measures in older Arabic-speaking adults with CRC. This is also one of the few studies to focus on the psychometric properties of the FRAIL scale, whereas most studies are designed to evaluate the scale’s predictive validity [58,59,60].

The FRAIL-AR scale showed acceptable internal consistency using the Kuder–Richardson Formula 20 (KR-20 = 0.80) [53]. This finding is consistent with the internal consistency reported for Saudi Arabian community-dwelling older adults in an Arabic FRAIL scale validation study (α = 0.79) [22]. In contrast, the internal consistency of the FRAIL-AR scale is higher compared to the Chinese versions of FRAIL (KR-20 ranging from 0.485 to 0.67) [19,61], the Japanese version (KR-20 = 0.32) [62], the Indonesian version (α = 0.67) [63], and the Brazilian Portuguese versions (KR-20 ranging from 0.447 to 0.53) [64,65]. These discrepancies might be attributed to linguistic and cultural differences in the perception of frailty, social inequalities impacting access to healthcare, or methodological differences between studies [66].

The correlation coefficient between the individual items of the scale and the overall Arabic FRAIL scale was *r* = 0.48 to 0.71. These results indicate that each item contributes substantially to the scale’s overall measurement of frailty. Our findings are relatively close to the correlations reported in a previous Saudi Arabian validation by Al Qahtani and Nasser (*r* = 0.44 to 0.69) [22]. This finding is also reported for an Indonesian version that reported correlation coefficients between the total score and individual items (*r* = 0.32 to 0.810) [63]. Moreover, our findings are relatively similar to those of Rosas-Carrasco et al. [21] and Susanto et al. [67], who found a significant correlation between four of the five scale domains and the total score (*r* = 0.41 to 0.74 and r = 0.39 to 0.82, respectively). However, these studies also reported a weaker correlation for “illness”. These discrepancies related to “illness” might be attributed to cultural differences in the perception of illness or methodological variations between the studies. Supporting this, Rosas-Carrasco et al. [21] suggested that comorbidities are not as closely related to the development of frailty as the other dimensions of the scale are. Furthermore, they noticed that several frailty screening tools such as the Fried criteria and the Groningen Frailty Indicator do not include them [68].

The test–retest reliability showed good stability for the total FRAIL-AR scale over a one-week interval (ICC = 0.89). Our findings align with the results of other validation studies, including the Indonesian version (ICC = 0.82) [63] and the Mexican Spanish version of the FRAIL scale (ICC = 0.82) [21]. However, FRAIL-AR has very good test–retest reliability compared with the Chinese version (ICC = 0.71) [19], the Brazilian version (ICC = 0.70) [64], and the previously validated Arabic version (ICC = 0.77) [22]. We found that the ICC of all four domains exceeded 0.8 in our study, except for the “ambulation” domain that had a lower ICC value (0.71) than the other domains in the FRAIL-AR scale. The moderate stability of the ambulation item can be attributed to its subjective, self-reported nature. In CRC patients, the perception of their ambulation ability often varies slightly from day to day due to fluctuating symptoms and fatigue. FRAIL-AR was evaluated for measurement variability and clinically meaningful changes, as recommended by Lexell and Downham [69]. The measurement error for the overall FRAIL score was 0.59 points, which seems acceptable and similar to that of the Japan Frailty Scale (SEM = 0.663) [55]. The SDC value reported in this study showed that an actual change in the overall frailty score of CRC participants could be achieved with 1.63 points, which is relatively lower than the SDC reported for the related Japan Frailty Scale (1.838) [55].

Our results support the expected negative weak correlations between the overall FRAIL-AR scale score/each individual items and physical function as measured using the EORTC QLQ-C30. This observed pattern of correlation may be attributed to the comprehensive nature of the physical function subscale of the EORTC QLQ-C30, which captures the full complexity of physical function, while a single item of the FRAIL-AR scale provides a more limited representation [70]. In the current study, the FRAIL-AR scale showed evidence of convergent validity as reflected by correlation with several frailty-related measurements, such as the TUG and 5xSTS scores. Our results show that the overall FRAIL-AR score demonstrated a strong positive correlation with TUG and 5xSTS, while each individual item demonstrated a moderate positive correlation with TUG and 5xSTS. This correlation is comparable to findings from an Arabic FRAIL scale validation study that reported a moderate correlation (ρ = 0.41) with TUG [22]. Furthermore, our findings align with previous studies reporting satisfactory correlations between overall FRAIL scale scores and physical function as measured using TUG and 5XSTS in older adults [21,22,61,62,71,72].

In the current study, the FRAIL-AR scale effectively differentiated frailty levels based on age, with participants aged ≥75 years having a significantly higher prevalence of frailty than those aged 65–74 years. Furthermore, older women with CRC had a significantly higher frailty prevalence compared to older men, which indicates a greater burden of frailty. This aligns with existing research indicating that increased age and being a woman are associated with a higher likelihood of frailty [73,74]. Several factors, such as age-related decline in physiological reserves and hormonal changes in women including reduced muscle mass and strength, contribute to increased frailty susceptibility—especially when compounded by stressors like cancer and its treatment and varying access to care [71,75].

The FRAIL-AR scale also showed discriminatory properties in identifying patients with frailty based on health status, demonstrating that those with more comorbidities were more likely to exhibit frailty characteristics. This result is consistent with previous studies reporting that patients with a severe CCI score (≥5) had a higher frailty rate (62%) than those with fewer comorbidities, further supporting the scale’s known-group validity [76,77,78]. The current study included 21 predefined hypotheses to examine validity. With 20 (95%) of these hypotheses accepted (Table 1), and confirming the pre-established ≥75% threshold, the FRAIL-AR scale is demonstrated to have very good validity and is considered a valid and appropriate screening tool for frailty screening in CRC patients.

The discrepancy related to weak or absent correlation of illness with total scale scores might be attribute to the explanation provided by Rosas-Carrasco et al. [21], who suggested that comorbidities are not as closely related to the development of frailty as they are to other dimensions of the scale. However, frail patients (FRAIL-AR > 3) in our cohort exhibited significantly higher comorbidities than non-frail patients (FRAIL-AR = 0–2); this directly addresses the uncertainty around the “illness” domain. Therefore, incorporating a multidimensional view of patient vulnerability, as captured by both comorbidity burden and frailty status, allows clinicians to better balance the potential benefits of aggressive therapy against the risks of toxicity and the competing causes of mortality. This patient-centered approach moves beyond chronological age to prevent both overtreatment of highly vulnerable patients and undertreatment of fit older adults, thereby optimizing the potential for positive outcomes and improved quality of life across the diverse older cancer patient population [79,80,81].

This study has some limitations. The age range of 65–75 years and recruitment from a single center limit the generalizability of the findings. Therefore, broader studies involving multiple centers and older populations, especially those over 75 who are often at increased risk of frailty, are needed to be nationally representative. The study’s focus on CRC patients might mean the FRAIL-AR scale’s validity varies for those with other chronic diseases. Therefore, more research is required to determine the scale’s performance across a range of chronic diseases. However, this study may add a simple alternative tool for frailty screening in the clinical practice of oncologists and healthcare providers. The cross-sectional design precludes examining the predictive validity of the questionnaires for adverse health outcomes such as risk of falls, readmission, and mortality. Finally, a limitation of the current study is the incomplete assessment of all psychometric properties according to the COSMIN guidelines—specifically, a formal factor analysis (structural validity). Thus, future validation work should consider this analysis to confirm the underlying factor structure of the FRAIL-AR scale. Despite these limitations, this study provides evidence that the FRAIL-AR scale is a reliable and valid screening tool for frailty in older Arabic-speaking colorectal cancer patients, offering a simple and practical alternative for use by oncologists and healthcare providers in clinical practice.

## 5. Conclusion

In conclusion, this study provides adequate evidence that FRAIL-AR is a reliable and valid tool for screening frailty in older Arabic-speaking colorectal cancer patients. Its established validity and reliability make it a practical and valuable tool for routine frailty screening in clinical settings and research studies.

## Figures and Tables

**Table 1:** Hypotheses and correlation coefficient for convergent (*n* = 18) and discriminate validity (*n* = 3).

	Hypothesis Statement	Hypothesized Correlation (Spearman)	Calculated Spearman Correlation Coefficient	Hypothesis Confirmed?
Convergent Validity Hypothesis (1–18)				
1–6	The overall scores of FRAIL-AR scales and its five domains (fatigue, resistance, ambulation, illness, and weight loss) would show a weak negative correlation with EORTC QLQ-C30 physical function score (*n* = 6)	(−0.20 to −0.39)	−0.15 to −0.38 **	Yes
7–12	The overall scores of FRAIL-AR scales and its five domains (fatigue, resistance, ambulation, illness, and weight loss) would show a moderate positive correlation with TUG scores (*n* = 6).	(0.40–0.59)	0.40–0.75 **	Yes
13–18	The overall scores of FRAIL-AR scales and its five domains (fatigue, resistance, ambulation, illness, and weight loss) would show a moderate positive correlation with 5xSTS scores (*n* = 6)	(0.40–0.59)	0.40–0.63 **	Yes
Discriminate validity hypotheses (19–21)		Hypothesized Test Result (*p*-value)	Calculated *p*-value	
19	The FRAIL-AR scores would be significantly greater among the participants ≥ 75 years with CRC compared to those aged 65–74 years.	*p* < 0.05	*p* < 0.05 *	Yes
20	Older CRC patients would have higher FRAIL scores for women than for main	*p* < 0.05	*p* < 0.05 *	Yes
21	Among older adult patients with CRC, those with a severe CCI score (≥5) demonstrated greater FRAIL-AR scores in comparison to those with a moderate CCI (3–4).	*p* < 0.05	*p* < 0.05 *	Yes

** Correlation is significant (*p* < 0.01, Calculated Spearman correlation coefficient), * Correlation is significant at (*p* < 0.05, calculated), TUG, Timed Up and Go, 5xSTS, Five Times Sit-to-Stand, CCI, Charlson Comorbidity Index. EORTC QLQ-C30: European Organisation for Research and Treatment of Cancer Quality of Life Questionnaire-Core 30 (version 3.0).

**Table 2:** Sociodemographic and Clinical Characteristics of Participants (*n* = 137).

Variables	Total (*n* = 137)	60–74 yrs (*n* = 101)	Age ≥ 75 yrs (*n* = 36)
Age, yrs Mean (SD)	68.04 ± 6.99	64.78 ± 4.53	77.19 ± 3.83
Gender Female Male	51(37.20%) 86(62.80%)	39(38.60%) 62(61.40%)	12(33.30%) 24(66.70%)
Marital status Married Single/Divorce/Widowed	103(75.20%) 34(24.80%)	82(81.20%) 19(18.80%)	21(58.30%) 15(41.70%)
Education Primary school Intermediate/Secondary school University/higher degree	56(40.90%) 36(26.30%) 45(32.80%)	37(36.60%) 28(27.70%) 36(35.70%)	19(52.80%) 8(22.20%) 9(25.00%)
Employment status Employed/self-employed. Unemployed/retired	38(27.70%) 99(72.30%)	29(28.70%) 72(71.30%)	9(25.00%) 27(75.00%)
Residential place Urban Rural	98(71.50%) 39(28.50%)	29(28.70%) 72(71.30%)	10(27.80%) 26(72.20%)
BMI (kg/m^2^) <25 ≥25	59(43.10%) 78(56.90%)	47(46.50%) 54(53.50%)	12(33.30%) 24(66.70%)
Smoking Yes No	29(21.20%) 108(78.80%)	10(9.90%) 91(90.10%)	19(52.80%) 17(47.20%)
Cancer type Colon Rectal	90(65.70%) 47(34.30%)	78(77.20%) 23(22.80%)	12(33.30%) 24(66.70%)
Tumor stage I–II III–IV	62(45.26%) 75(54.74%)	51(50.50%) 50(49.50%)	11(30.60%) 25(69.40%)
Cancer duration <3 years ≥3 years	99(72.30%) 38(27.70%)	90(89.10%) 11(10.90%)	9(25.00%) 27(75.00%)
Type of intervention Surgery Chemotherapy Radiotherapy Surgical + chemotherapy Chemo + radiotherapy Surgical + chemo + radiotherapy	20(14.60%) 17(12.40%) 11(8.00%) 28(20.40%) 6(4.40%) 55(40.10%)	19(18.80%) 14(13.90%) 11(10.90%) 23(22.80%) 4(4.00%) 30(29.70%)	1(2.80%) 3(8.30%) 0 5(13.90%) 2(5.60%) 25(69.40%)
Co-morbidities Mild risk (1–2) Moderate risk 3–4 Sever risk ≥ 5	0 80(58.40%) 57(41.60%)	0 50(49.50%) 51(50.50%)	0 30(83.30%) 6(16.70%)

BMI: body mass index, SD: standard deviation, *n*: number, yrs: years.

**Table 3:** Internal consistency (*n* = 137) and test–retest reliability (*n* = 30) of the FRAIL-AR Scale among participants with colorectal cancer.

Domain	Item-To-Total Spearman Correlation (r) (95% CI)	*α*	ICC_2.1_ (95% CI)
Fatigue	0.67(0.49–0.71) **	-	0.85 (0.71–0.93) ^‡^
Resistance	0.71(0.58–0.76) **	-	0.89 (0.79–0.95) ^‡^
Ambulation	0.71(0.61–0.78) **	-	0.71 (0.48–0.85) ^‡^
Illnesses	0.48(0.45–0.71) **	-	0.94 (0.87–0.97) ^‡^
Loss of weight	0.60(0.50–0.79) **	-	0.80 (0.62–0.90) ^‡^
Overall FRAIL score	-	0.80 (0.73–0.85)	0.89 (0.77–0.94) ^‡^

** Correlation significant at the (*p* < 0.01), ^‡^ Correlation is significant at the (*p* < 0.01) for Intraclass Correlation Coefficient, ICC_(2.1)_: intraclass correlation coefficients two-way random-effects model on absolute agreement, CI: Confidence interval, α: represents Kuder-Richardson formula 20 (KR-20).

**Table 4:** Convergent Validity of the FRAIL-AR Scale with EORTC QLQ-C30 physical function scale, TUG and 5xSTS scores (*n* = 137).

FRAIL-AR Scale	Fatigue	Resistance	Ambulation	Illnesses	Weight Loss	Overall FRAIL Score
	r_s_	r_s_	r_s_	r_s_	r_s_	r_s_
EORTC QLQ-C30 Physical function scale	−0.24 *	−0.29 *	−0.32 *	−0.15 *	−0.22 *	−0.38 *
TUG score	0.50 **	0.47 **	0.49 **	0.53 **	0.40 **	0.75 **
5xSTS score	0.40 **	0.43 **	0.43 **	0.42 **	0.46 **	0.63 **

r_s_: Spearman correlation coefficients, * statistically significant (*p*-value ≤ 0.05), ** statistically significant (*p*-value ≤ 0.01). TUG: Timed Up and Go, 5xSTS: Five Times Sit-to-Stand. EORTC QLQ-C30: European Organisation for Research and Treatment of Cancer Quality of Life Questionnaire-Core 30 (version 3.0).

**Table 5:** Known-Group Validity of the FRAIL-AR Scale Based on age, gender, and comorbidities (*n* = 137).

FRAIL-AR Scale	Age Group (Years) ^^^		Gender ^^^		CCI Scores ^^^	
	60–74 (*n* = 101)	≥75 (*n* = 36)	Male (*n* = 86)	Female (*n* = 51)	Moderate 3–4 (*n* = 80)	Severe ≥ 5 (*n* = 57)
Robust (score = 0)	39 (38.60%)	7(19.44%)	31(36.05%)	12(23.50%)	26(32.50%)	11(19.30%)
Prefrail (score 1–2)	29(28.70%)	8(22.22%)	25(29.07%)	15(29.40%)	25(31.30%)	15(26.30%)
Frail (score ≥ 3)	33(32.70%)	21(58.34%) **	30(34.88%)	24(47.10%) *	29 (36.20%)	31(54.40%) *
Effect size (95%CI) (Overall FRAIL score)	0.38 * (0.1–0.67)		0.45 * (0.15–0.84)		0.49 * (0.1–0.79)	

CCI: Charlson Comorbidity Index, ^^^ chi-square (*χ^2^*) analysis, * statistically significant (*p*-value ≤ 0.05), ** statistically significant (*p*-value ≤ 0.01).

## Data Availability

The data presented in this study are available upon request from the corresponding author. Owing to participant privacy and ethical restrictions related to the sensitive nature of patient data in our study, the raw data cannot be made publicly available. However, we are willing to provide the minimal dataset necessary for validation upon reasonable request, subject to compliance with ethical and privacy regulations.

## Abbreviations

The following abbreviation are used in this manuscript:

CRC	Colorectal Cancer
FRAIL-AR scale	Arabic FRAIL scale
TUG	Timed Up and Go Test
5xSTS	Five Times Sit-to-Stand Test (used in Methods and Results)
KR-20	Kuder-Richardson formula 20
ICC	Intraclass Correlation Coefficient
CI	Confidence Interval
KFSHRC-Jeddah	King Faisal Specialist Hospital Research Center in Jeddah
CCI	Charlson Comorbidity Index
EORTC QLQ-C30	European Organization for Research and Treatment of Cancer Quality of Life Questionnaire-Core 30
QOL	Quality of Life
